# Intra-Company Crowdsensing: Datafication with Human-in-the-Loop

**DOI:** 10.3390/s22030943

**Published:** 2022-01-26

**Authors:** Jaroslaw Domaszewicz, Dariusz Parzych

**Affiliations:** 1 Institute of Telecommunications, Faculty of Electronics and Information Technology, Warsaw University of Technology, 00-665 Warsaw, Poland; 2 Faculty of Management and Center for Innovation and Technology Transfer Management, Warsaw University of Technology, 02-524 Warsaw, Poland; Dariusz.Parzych@pw.edu.pl

**Keywords:** mobile crowdsensing, participatory sensing, human sensor, human-computer interaction, context awareness, IoT, digital/human work configuration, internal crowdsourcing, organizational aspects

## Abstract

Every day employees learn about things happening in their company. This includes plain facts witnessed while on the job, related or not to one’s job responsibilities. Many of these facts, which we call “occurrence data”, are known by employees but remain unknown to the company. We suppose that some of them are valuable and may improve the company’s situational awareness. In the spirit of mobile crowdsensing, we propose intra-company crowdsensing (ICC), a method of “extracting” occurrence data from employees. In ICC, an employee occasionally responds to sensing requests, each about one plain fact. We elaborate the concept of ICC, proposing a model of human-system interaction, a system architecture, and an organizational process. We position ICC with respect to related concepts from information technology, and we look at it from selected organizational and managerial viewpoints. Finally, we conducted a survey, in which we presented the concept of ICC to employees of different companies and asked for their evaluation. Respondents positive about ICC outnumbered skeptics by a wide margin. The survey also revealed some concerns, mostly related to ICC being perceived as another employee surveillance tool. However, useful and acceptable sensing requests are likely to be found in every organization.

## 1. Introduction

Consider energy consumption in a typical office building. Energy gets wasted by lights lighting empty spaces, thermostats set to heat stairways to room temperature, or air conditioners cooling empty rooms. These facts are easy to spot, but office workers do not recognize the problem as theirs and do not pay attention to them. We argue that there are many areas where plain facts, related to how the company functions, go unnoticed or overlooked. They include security, customer behavior, and work safety, to name a few. And there is an opportunity here; knowing these facts may improve the company’s situational awareness and operation.

A seemingly obvious solution is to instrument space with sensors. The approach is consistent with current trends, such as the Internet-of-Things (IoT), but it does have disadvantages. The sensors need to be deployed and maintained. Some sensor data processing tasks are quite hard for computers to perform (e.g., image understanding). Carbon footprint, used batteries, and electronic waste contribute to an environmental impact. On top of that, ubiquitous sensing infrastructure may create a panopticon-like environment, with employees under constant surveillance.

Our approach is not meant to replace, but to complement IoT. It relies on humans; the sources of the facts are the company’s own employees, who contribute by responding to *sensing requests*. Each sensing request is about a plain fact, which should be known to an employee by virtue of their performing regular activities at the company. A software system distributes the requests so that no employee gets overloaded. Notably, it is up to the recipient whether to respond or not. We call the above system *intra-company crowdsensing*. It is *crowdsensing* in that it relies on a potentially large population (i.e., all employees) to “sense.” It is *intra-company*, as it is limited to the employees and does not reach out to the general public.

This paper makes three contributions. First, we put forward the thought that some plain facts, tacitly known by employees, may, when acquired, form a potentially valuable resource. We name the resource “occurrence data” and list some of its characteristics. Second, we contribute the concept of intra-company crowdsensing (ICC), a method to extract occurrence data from employees. ICC is software-driven and can be considered a novel digital/human work configuration [1]. We elaborate ICC, proposing a human-system interaction model, a system architecture, and an organizational process. We position ICC against the background of information technology and look at it from several managerial perspectives. Third, we offer a survey-based evaluation of ICC. We present the method to employees of different companies and ask for their opinions and suggestions. In our coverage of ICC, we gather our own proposed solutions, applicable existing research, and views of our respondents, all to provide value to those interested in pursuing this exploration further. In particular, we uncover ICC-related challenges that deserve most attention.

As the name implies, the foundational concept for ICC is crowdsensing, and, more specifically, mobile crowdsensing (in its participatory version, where a person performs an action to contribute a piece of data). Our work belongs to the line of research that considers mobile crowdsensing, which relies on humans, as complementary to or synergistic with IoT, which relies on embedded nodes and other computational resources. Here we mention some examples of this approach. A recent survey [2] reviews promising studies that use participatory mobile crowdsensing as a means of interaction between the users of an office building and its BMS (building management system). In [3], the detection of events in urban areas was studied. The paper explores the benefits of a hybrid system that combines mobile crowdsensing (in its opportunistic version, where sensing is done by a mobile device without the direct involvement of its owner) with a network of fixed sensors deployed in an area of interest. In [4], combining different actors, i.e., humans, robots, and environmental sensors, into a single sensing system is termed “heterogeneous crowdsensing”; complementary sensing powers and kinds of intelligence exhibited by the different “participants” in heterogeneous crowdsensing is highlighted. In [5], visual data from a city, obtained via participatory crowdsensing, was fed into the city’s 3D digital twin, thus making the twin more aligned with reality. In [6], crowdsensing is seen as a new generation of wireless sensor networks. The paper reviews techniques to produce much needed location information for carried or worn mobile sensors, which work on top of heterogeneous communication technologies.

While ICC can essentially be considered a special case of participatory mobile crowdsensing, two clarifications are in order. First, we explore participatory sensing in the unique context of a company. Being “intra-company” gives rise to a number of assumptions, problems, and opportunities, which do not exist in the “public” case. In that sense, the paper seems to open a new niche in mobile crowdsensing. Second, while ICC does indeed rely primarily on mobile devices, it may use other devices as well; sensing requests may be delivered via any personal device operated by an employee (e.g., a laptop or desktop), or via shared stationary terminals dedicated to specific locations. In that sense, crowdsensing is extended beyond relying solely on mobile devices; data acquired from human sensors matter more than the kind of devices used. We offer more information on how our work relates to mobile crowdsensing (and similar concepts) in Section 6.

From now on, we use the words “user” and “employee” interchangeably: in terms of user interaction, the employee who handles a sensing request is the primary user of an ICC system. In addition, we use “company” and “organization” interchangeably (we could use “enterprise” as well). Importantly, ICC is envisioned as applicable in *any* organization, not necessarily a business-oriented or industrial one.

In Section 2, we introduce occurrence data. In Section 3, we define intra-company crowdsensing and provide examples of sensing requests. In Section 4, we cover human-system interaction and user experience issues. In Section 5, we propose a generic ICC system architecture. In Section 6, we discuss how ICC relates to existing approaches in information and communication technologies (ICT). In Section 7, we look at ICC as an organizational process, and, in Section 8, we continue with an organizational and managerial outlook. In Section 9, we present the survey and its results. Section 10 concludes the paper.

## 2. Occurrence Data as Company’s Overlooked Resource

Every day each employee learns about what is going on in their company. This may include complex insight, such as understanding the company’s market position derived from sales figures. However, much of what an employee learns are plain facts they simply witness while performing regular job activities or just by “being there” (at the company’s premises, with a customer, etc.). Notably, the facts need not be related to the employee’s job responsibilities. We use the term “occurrence data” to denote company-relevant plain facts (“occurrences”) an employee learns by doing their job at the workplace.

A tentative characterization of occurrence data items may look as follows:

1. They are specific and unambiguous.
2. They are heterogeneous and cover many areas, even for a single employee. One person may know very different things about the company.
3. They may differ from person to person, even if the two hold the same positions and work at the same place. Different employees may be attuned to different things.
4. They are rarely at an employee’s center of attention. They come from the working environment naturally and implicitly. An employee may consider them insignificant or “get used” to them, thus keeping them at the periphery of attention.
5. Many of them end up in short-term memory and may be quickly lost if not captured.
6. Due to their simplicity, they should be easy to store and process by a machine (possibly after being combined with other kinds of data).

An employee knows about many occurrences, and occurrence data may be of value to the company. However, a large part of the data is not captured; it never enters a reporting system or data repository. Just like there is the problem of unshared knowledge [7], there is the problem of unshared occurrence data.

## 3. Intra-Company Crowdsensing: Definition and Examples

We propose intra-company crowdsensing (ICC) as a continuous, software-driven process of acquiring occurrence data from a company’s employees. It consists in regularly delivering sensing requests and collecting responses. Each sensing request concerns a plain fact, occurring now or in the recent past; it is not about an opinion, interpretation, or judgment. It is phrased as a closed-ended question or asks for a number. Once a request is delivered, it is up to the recipient whether to respond or not.

ICC is best explained by examples of sensing requests, as in Table 1. The examples are grouped into ICC “application areas,” as diverse as energy and water conservation, customer intelligence, security and cybersecurity, occupational safety and health-promoting work habits, sociometry and the efficiency of work activities, and, finally, the availability of workplace facilities and supplies. If needed, a sensing request in Table 1 is accompanied by a brief annotation on its context of use or recipient (in italics). Some of the examples may be used at any company, while others are company specific. Similarly, some sensing requests may be directed to most (or all) employees, while others are tailored to a specific role. In general, sensing requests should be designed for a given company and then for different roles its employees play.

Certainly, not all potential ICC application areas are represented in Table 1. Quite a few examples belong to the office and building domain; one can certainly find more from construction, manufacturing, logistics, and the like. A company-wide ICC system can include sensing requests from multiple areas and domains, and the number of requests belonging to any of them may vary from one to many. Furthermore, both the ICC application areas and their sensing requests may change over time.

## 4. User Interaction Model and User Experience Issues

For ICC to be successful, employees need to accept it and cooperate. Accordingly, the overall user experience is crucial. This is why ICC, as defined above, works in the pull mode: it is the system that triggers an action by delivering a sensing request. The push mode, i.e., contributing on one’s own initiative, would place more burden on an employee. For the same reason, we restrict our attention to voluntary participation. Here we describe an ICC interaction model and touch upon some user experience issues.

### 4.1. ICC Terminals and Request Delivery Modes

We consider two kinds of terminals used for the delivery of sensing requests to a user: (a) personal terminals and (b) point of interest (POI) terminals. In either case, the terminal displays a sensing request and allows the user to enter a response. Personal terminals are primarily mobile devices, but they may also include laptops or desktops “owned” by an employee. A user interface like that of a messenger application may be used with a personal terminal. A less typical example of a personal terminal, designed for extreme conditions, would be an ICC-tailored wristband.

We envision a *POI terminal* to be a compact, dedicated, standalone device deployed in some area. The terminal features a minimalistic user interface: a tiny screen and some buttons. On top of that, the POI terminal can alert people nearby, via blinking or beeping. The alerting starts when a new sensing request becomes available and stops after some time to avoid excessive disruption. The POI terminal is primarily meant to be deployed near an object or location of interest. However, it may be used to deliver assorted sensing requests.

In line with our classification of terminals, we distinguish two delivery modes. With *personal delivery* one delivers to a specific person. Thus, if needed, one can reach employees who play specific roles matching the sensing request. In addition, personal delivery mitigates the diffusion of responsibility [8]. With *POI delivery* one usually wants to reach anybody near a point of interest. POI delivery ensures anonymity and the lack of pressure on employees.

Personal delivery can be context-aware [9], e.g., to avoid interrupting an employee when they are focused. Consider a bus driver. A sensing request should not be delivered when the bus moves, but it probably could be delivered at a bus stop (a condition easy to detect). Another reason for context-awareness (actually, location awareness) is to emulate POI delivery with personal delivery; a sensing request may be delivered to a person when they happen to be at a location of interest.

### 4.2. Receiving and Reacting to Sensing Request

**Figure 1:**
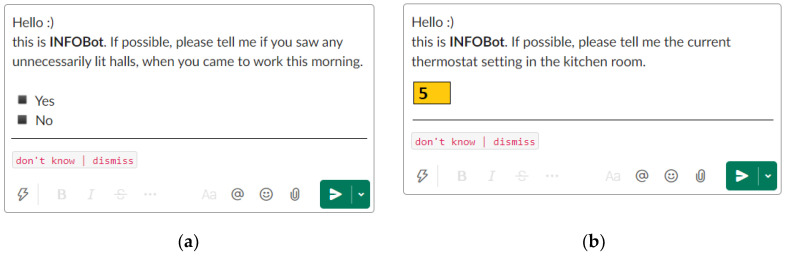
Possible rendering of sensing requests, as presented to a user: (**a**) a closed-ended question and (**b**) a question about a number; based on Slack [10].

Receiving and handling a sensing request is the key use case in ICC. The request is a brief question about a plain fact. As shown in Figure 1, the question may be closed-ended (i.e., provided with possible answers), or it may ask for a number. Two generic buttons, “don’t know” and “dismiss”, are always available.

A user who received a sensing request may react in different ways. The simplest one is to *disregard* the request, i.e., to do nothing, abstaining from any interaction with the terminal. In that case, after some expiry time, the request is withdrawn. The user may also explicitly *dismiss* the request using the dedicated buttons. As participation is voluntary, the user should be aware that disregarding and dismissing a request are as good reactions as any other.

The user may also decide to *respond* to the request, by picking one of the presented answers or entering a number. The response should come either off the top of the user’s head or result from a brief check of a nearby place. In the latter case, the user may need to move to the place in question, look around, and go back (e.g., for a personal delivery, when the user is at their desk), or just to look around (e.g., for a POI delivery, when the user is already at the right place).

### 4.3. System Intrusiveness and Load Balancing

The effort of handling a sensing request should not exceed that of answering an SMS or replying to an instant message. However, every sensing request introduces some disruption, which may lower the efficiency of doing primary tasks or cause a negative user reaction. This is especially the case for personal delivery when the recipient “owns” the request. We assume that ICC includes a load balancing mechanism. It is a technique of allocating requests to users so that no single user gets overwhelmed by too many of them delivered in a short time. In the proposed architecture of the ICC system (see below), we introduce a component responsible for load balancing.

### 4.4. Self-Incrimination and Loss of Privacy

So far, we have described the ICC user interaction model without paying attention to what a sensing request is about. Many requests are quite “neutral” (e.g., “How many customers are currently looking at self-help books?”). Sometimes, however, responding to a sensing request may reflect badly on the person who answers or their colleagues. An honest response may imply that somebody is negligent (“Can you see any dangerous tools left unattended?”) or has poor work habits (“Did your last meeting end with action items?”). And even if a response itself is not negative, it may lead to undesirable loss of privacy. For example, responding “yes” to “Was the company parking lot full when you arrived today?” may reveal that the employee comes to work later than most (while still within company-set limits). In short, there is the danger that ICC may be perceived as a “big brother”; people may reject it because they do not want to reveal things about themselves or act as informers.

It is an open issue how to ensure that people are not discouraged by incriminating responses or the loss of privacy, inherent in some (not all) sensing requests. Sometimes, anonymization is an option. It is not always possible, but, notably, POI delivery guarantees anonymity by design. Another approach is for the software to raise the level of abstraction of response-derived information. This way, ICC may be used to spot systemic problems rather than specific instances of “wrongdoing.” In some cases, employees will understand that concerns related to incrimination and privacy should come second to bigger issues. There are areas where not following procedures may lead to disastrous consequences, as in work safety and accident avoidance. In those cases, ICC may facilitate a kind of internal whistleblowing [11]. Yet another approach may have to do with an explicitly stated organizational culture that promotes openness, awareness, and continuous improvement rather than punishment. Finally, an employee may always decide not to respond.

## 5. Generic ICC System Architecture

In this section, we outline a high-level ICC system architecture and some lower-level details on how the system could be implemented. These are not the only possible choices; one can make different ones within the general ICC concept presented above. We use both the architecture and the details to exemplify and clarify the concept, not to limit it.

### 5.1. ICC as Part of Context-Aware System

**Figure 2:**
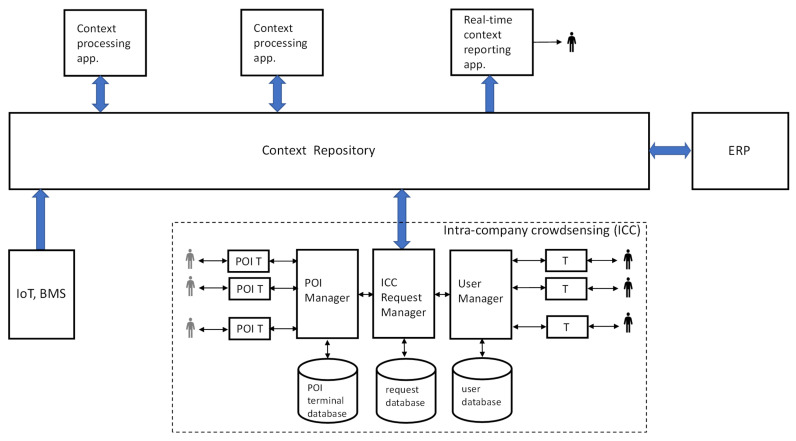
An ICC sub-system integrated with a context-aware system.

The architecture, shown in Figure 2, includes various sources of data about the state of the environment, not just ICC. These may be physical (non-human) sensors, such as those found in IoT instrumentation or a building management system (BMS). We use the term “context” (as in “context-aware computing”) to refer collectively to all such data. The intention is to treat all context uniformly, no matter whether a data item comes from a physical sensor or a human. Accordingly, all context data end up in the context repository, a core element in any well-engineered context-aware system [12].

Figure 2 also shows some applications that use the context. One of them may generate higher-level insight from low-level context data. Such an application, called a logical sensor, is a place for possibly sophisticated AI, big data, or reasoning algorithms. Another application may drive actuators (not shown) to change the state of the environment. Yet another performs real-time context reporting to a human via a dashboard or alerts. As we refer to a company setting, Figure 2 shows integration with the company’s ERP (enterprise resource planning) system. Such integration is certainly desirable, but the system shown in Figure 2 could deliver value without a connection to ERP.

In summary, Figure 2 shows a complete context-aware system, of which ICC is a part. One could build a system like this around an existing context-awareness platform, e.g., FIWARE [13]). Of course, ICC must then obey the data model used by the platform’s context repository. Thus, introducing ICC does not require building the system shown in Figure 2 from scratch. Instead, it is more about integrating an ICC sub-system with an infrastructure already in place.

### 5.2. ICC Databases: Sensing Requests, Users, POI Terminals

The ICC sub-system itself is driven by a request database, user database, and POI terminal database. All the three databases include profiles (attributes) and histories (assorted timestamped events) for their respective entities. The request database stores sensing requests used by the system. The profile of a sensing request may include the following attributes: (a) the text of the request, along with possible responses (as displayed on an ICC terminal), (b) a target location (optional), (c) a list of target roles (optional), (d) a request delivery schedule (optional), (e) request delivery context triggers (optional), (f) an oversampling factor, and (g) a response metadata pattern.

The target location, if applicable, indicates a place on the company’s premises where an object or area of interest is (e.g., consider a sensing request referring to the thermostat in the kitchen room). It maps a request to the logical organization of the company’s space. A request with a target location is called *location-targeted*.

The list of target roles specifies to whom the request may be delivered. A target role may include a division and position within the division. Note that here, one does not identify people, just roles. Again, the target roles are included only if applicable (e.g., if the request is about the number of customers in some store area, it should probably be delivered to a salesperson and not to the store manager). A request with one or more target roles is called *role-targeted*. Notably, the request database is agnostic as to the workforce; determining employees that play any of the target roles requires access to the user database, as described below. Note that being role-targeted is independent of being location-targeted (all four combinations are possible).

The delivery schedule, included for requests with *time-triggered delivery*, specifies at what times, or how often, the request should be delivered to users. The other possibility is *context-triggered delivery*, where the request is delivered when some conditions, called context triggers, become fulfilled (e.g., when the outside temperature drops below zero). In general, delivering a request may be both time-triggered and context-triggered. The oversampling factor has to do with delivering one request to multiple users at a time to ensure the reliability of acquired data; it is the number of users from which the system seeks a response to the request at any given time. Finally, the response metadata pattern is a template for metadata added to a response when it is injected into the context repository.

The user database stores information about the company’s employees. An employee’s profile may include: (a) their role (e.g., the division and position), (b) the location they work at (e.g., a room number), (c) locations they have access to, (d) their working schedule, and (e) context prerequisites for delivery (optional). The last item specifies that a request can be delivered to the employee only if some employee-related context conditions are met (e.g., only if the employee is in the conference room). The POI terminal database stores information on POI terminals. A POI terminal’s profile may include its location, interaction capabilities (e.g., whether it can display an arbitrary request), etc.

Note the difference between a context trigger and a context prerequisite. The former belongs to the profile of a request and specifies that the request should be delivered when some environment-related contextual condition is met. The latter belongs to the profile of a user and specifies that no requests should be delivered to that user unless a user-related contextual condition is met.

### 5.3. ICC Components: Request Manager, User Manager, POI Terminal Manager

**Figure 3:**
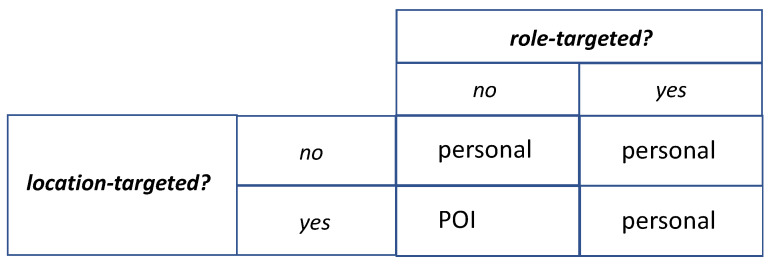
A possible mapping of different kinds of sensing requests to the delivery modes. Other mappings are possible, except for role-targeted requests, which require personal delivery.

The three major components of the ICC sub-system are the request manager (ReqMngr), user manager (UserMngr), and POI terminal manager (POIMngr). Each of them uses its respective database. ReqMngr detects when each sensing request should be delivered to users, initiates deliveries, and, having received responses, stores them in the context repository. To find out when to deliver, ReqMngr inspects the delivery schedule and context triggers (checking if the context triggers’ conditions are met is done by consulting the context repository). It may also take the request history into account. When a request is to be delivered, ReqMngr decides on the delivery mode. A possible mapping of different kinds of sensing requests to the delivery modes is shown in Figure 3. Then the request is passed to UserMngr for personal delivery or to POIMngr for POI delivery.

The user manager (UserMngr), having received a request from ReqMngr, picks the user(s) to whom the request should be delivered. It does so by inspecting the request’s target roles and location (if any) and consulting the user database. First, UserMngr finds all eligible users: (a) for a role-targeted request, a user’s role in the organization must match at least one of the target roles, (b) for a location-targeted request, a user must have access to the target location. If the request is neither role-targeted nor location-targeted, all employees are eligible.

Next, UserMngr finds the most appropriate users among the eligible ones. For each, it checks their working schedule and history. It finds out if another request delivery would not overload the employee (load balancing). For a location-targeted request, UserMngr estimates how long it would take the user to get to the location. The user manager also consults the context repository to check if the user’s current context satisfies their contextual prerequisites. Based on these factors, UserMngr picks the recipients (ideally, as many as the request’s oversampling factor). Thus, UserMngr is responsible both for delivery to the right employees and the overall user experience. In the end, UserMngr displays the request on the recipients’ personal terminals and collects responses (or indications of dismissal or disregard). These are passed to ReqMngr.

The POI terminal manager (POIMngr), having received a request and its target location from ReqMngr, finds POI terminals with matching locations. It picks some of them to display the request. Then POIMngr collects responses and passes them to ReqMngr. The latter, having received the responses from either of the two other managers, stores them (along with the request’s metadata) in the context repository.

Note that introducing the manager components allows us to separate concerns. The first one is *when* to deliver a request; this is the responsibility of the request manager. The other concern is *how* to deliver it in the best way (i.e., which users and POI terminals to involve); this is the responsibility of the other two managers. Also, note that a company-wide messaging application (e.g., Slack [10]), if deployed, could be used to handle user interaction. Then the ICC sub-system would conveniently take advantage of the existing communication infrastructure.

### 5.4. Notes on Selecting Delivery Modes

First, note that the POI delivery, as open to any user, is unsuitable for role-targeted requests; for them, one must use personal delivery. Next, we assume that location-targeted requests that are not role-targeted are passed for POI delivery (Figure 3). This is a natural choice, as the main reason for having POI terminals is to cover a specific location. However, if a POI delivery does not produce a response (maybe nobody is nearby the selected POI terminal), ReqMngr may try personal delivery; then, a responding employee needs to move to the location to inspect it.

We also assume, somewhat arbitrarily, that requests that are neither location-targeted nor role-targeted are passed for personal delivery (Figure 3). If a personal delivery does not produce a response (maybe all recipients have dismissed the request), ReqMngr may try POI delivery. In that case, the fact that POI terminals have specific locations does not matter; they become a means to deliver a location-neutral request.

Combining the above, it follows that an ICC sub-system can work without POI delivery, i.e., without POI terminals. If there are no role-targeted requests, it can work without personal delivery. In that case, all requests (location-targeted or not) would be delivered via POI terminals, with the advantages of anonymization and lack of pressure on employees.

### 5.5. Implementation Challenges

The total amount of data handled by an ICC sub-system is limited by the lowest throughput stage in the process, namely a human. Therefore, we expect that, even for large organizations, ICC will not be demanding in terms of extra high-performance computational resources, storage, or communication facilities.

The challenges in implementing ICC lie in modeling, algorithms, and integration. Let us assume that one wants to build a reusable ICC sub-system, i.e., one that can be used in different companies, after being customized for each one. Then such a platform should be based on a meta-model that allows one to develop and enter a model of a specific company, as part of the customization. The model of a company would include its organizational structure (e.g., its divisions and their relationships), the structure of its workforce (e.g., the positions or roles that can be held by its employees), and the composition and topology of its facilities (e.g., what logical locations are available, and how far away they are from one another). Once the platform is equipped with the model of the company, it can be populated with individual employees and sensing requests. The modeling challenge belongs to knowledge engineering and may use ontologies. For example, one can start with The Organization Ontology, available as a W3C recommendation [14].

Algorithms are needed to drive the operation of the request manager, user manager, and other components. For example, the request manager needs an algorithm to decide on the most promising delivery modes and an algorithm to detect fake responses to sensing requests with an oversampling factor greater than one. The user manager needs a load balancing algorithm and an algorithm that finds the distances between employees and target locations.

Finally, the integration challenge concerns interoperability of the ICC platform with the context repository and other pre-existing components of a company’s IT infrastructure. For example, it may require the development of a data model translation plug-in, so that data acquired with ICC may be stored in the context repository, such as the FIWARE Context Broker [13].

## 6. Related Concepts from ICT

Here we consider links between ICC and concepts usually elaborated within ICT (information and communications technologies). We briefly present each related concept and pinpoint features that make ICC distinct.

### 6.1. Mobile Crowdsensing, Citizen Sensing, People-as-Sensors

In mobile crowdsensing [15,16], many people contribute data via their mobile devices. A data item may come from a device’s sensor without human involvement (opportunistic sensing), or it may result from a user’s action (participatory sensing) [17]. Mobile crowdsensing has been applied in several domains, e.g., smart city [18,19]. Typically, participants come from the public at large (e.g., they are city dwellers). “Citizen sensing” [20,21], “citizen science” [22,23], “citizen as sensors” [24], and “people as sensors” [25] are all terms with considerable overlap with mobile crowdsensing. They imply the involvement of “citizens” rather than the employees of a company.

In ICC, most sensing requests are likely to be delivered via mobile devices. This makes ICC almost a variety of participatory mobile crowdsensing. It is a variety clearly distinguished by several characteristics. First, in ICC, potential participants do not come from the public. They are “insiders” who share a wealth of intra-company knowledge, operating procedures, values, objectives, etc.; the questions that can be asked of such participants and the way to communicate with them should be different. Second, the crowd of employees is structured and heterogeneous in terms of positions (roles). Accordingly, some sensing requests, rather than being directed to everybody (as in the case of a “flat” public crowd), can be role targeted. Third, while a mobile crowdsensing campaign usually covers a single issue, ICC may span multiple application areas within a single company (as shown in Table 1). Finally, in ICC, one need not be restricted to mobile devices but may also apply laptops, desktops, or, most notably, dedicated stationary POI terminals. The last characteristic means that ICC is not entirely subsumed by mobile crowdsensing.

Mobile crowdsensing has been recently advocated in the context of industry, and, more specifically, Industry 4.0 [26,27,28]. Most employee-related use cases covered in those papers fall under opportunistic sensing, i.e., using physical sensors embedded in employees’ mobile devices (not the case in ICC). “Subjective” data provided by humans, “subjective assessments,” “human knowledge,” and “integrated human wisdom” are also mentioned in [26,27,28], with at least a part of this input meant to come from employees (the other part coming from customers). These ideas are close to ICC but are put forward in general terms. Moreover, ICC is not limited to industrial, or even business-oriented, organizational settings.

Participatory sensing by employees can be traced in the experiment presented in [29]. There, if physical sensors detect lights switched on in an empty room, a so-called “actuation request” is delivered to a nearby employee. The employee is asked to check if the light is indeed on and, if so, to switch it off. Asking the employee to check the light may be interpreted as a “sensing request” embedded in the actuation request. However, the main means of sensing used in [29] is via physical sensors.

In summary, while there exists an extensive body of research devoted to a “crowd” contributing data, there seems to be little on this idea in the context of any company or organization, where employees do participatory sensing. At the same time, some results on mobile crowdsensing can certainly be applied to ICC.

### 6.2. Human Computation

Human computation is defined by two conditions: (a) the problem fits the general paradigm of computation, and as such might someday be solvable by computers, and (b) human participation is directed by a computational system or process [30]. Evidently, ICC satisfies both conditions quite well. First, in principle, data from ICC could also be obtained from a cyber-physical (computing) system, using sensors and algorithms. Second, the delivery of sensing requests is managed by the ICC (computer) system, which thus directs the human participation.

Human computation is often applied to tasks that are easy for humans, while hard or time-consuming for computer algorithms (e.g., image labeling). This is certainly the case for many ICC sensing requests; responding to them may require a high-level understanding of the situation witnessed by an employee. ICC is a kind of human computation, and some principles of human computation may apply to ICC.

### 6.3. IoT, Cyber-Physical-Human Systems, Human-in-the-Loop Cyber-Physical Systems

IoT nodes with sensors may feed the context repository with data of all kinds, in real time. We envision ICC, not as competing with IoT-based sensing, but as complementary to it. Both serve the same purpose, with different means (physical vs. human sensors). This is reflected in Figure 2, where data from both IoT and ICC end up in the context repository, stored in the same format. The two technologies can coexist within a single company. Further, we see IoT and ICC, to some extent, as interchangeable. ICC can be used instead of IoT when physical sensors have not yet been deployed, when they are expensive to deploy, or when sensing relies on understanding a situation (i.e., when humans are better than computers). As more physical sensors are deployed, ICC can be scaled down accordingly.

One could argue that data acquired from people are fundamentally less reliable than those coming from physical sensors. However, this is more a matter of degree. In pervasive computing, assessing the quality of inherently imperfect context information has been an important issue for a long time [31]. Data coming from non-human sensors have been described as possibly incomplete, imprecise, ambiguous, inconsistent, and outdated [9]. Accordingly, various quality metrics, to be associated with every piece of context information, have been proposed. One may adopt a similar approach for data originating from ICC. Generally, we advocate treating ICC-originated data in much the same way as data coming from physical IoT sensors to the extent possible.

ICC has a human dimension, and it qualifies as a cyber-physical-social system (CPSS) [32] or a cyber-physical-human-system (CPHS) [33]. “Humans in CPSSs can be viewed as not only the service consumers but also the service providers” [32], a feature that ICC certainly exhibits. In [32], citizen sensing is presented as a key example of the role humans can play in CPSS. A CPHS participant model given in [33] may be used to enhance the ICC user database (see Section 5) to make better decisions in allocating role-based sensing requests to employees.

A term closely related to CPSS and CPHS is human-in-the-loop cyber-physical systems (HiTLCPS or HiLCPS) [34]. Responding to ICC sensing requests can be positioned within a HiTLCPS taxonomy [34] in two places: in sensing (“direct feeding the system with information”) and processing (e.g., understanding a scene an employee looks at).

### 6.4. Experience Sampling in Pervasive Computing

The experience sampling method (ESM) originates in psychology, but it is also used in pervasive computing to evaluate solutions with users [35,36]. ICC and ESM are similar in that participants are prompted to enter data while “in the field” [36]. The prompted user responds to a sensing request (in ICC) or fills out a short questionnaire (in ESM) [35]. In both methods, the user should be able to respond with ease. As the name implies, ESM is more about experiences, while ICC is about plain facts.

Some arguments in favor of ESM (e.g., its ecological validity) seem to apply, after minor changes, to ICC. In addition, some concerns (e.g., about the burden on the participant, a possible drop in motivation, or the quality of contributed data [36]) are similar. The common mode of operation gives rise to similar design issues. Examples include (a) how not to overload a user with interruptions, (b) how long to wait before deciding that a user has disregarded a prompt [35,36], or (c) whether to prompt in a context-aware way. One approach to motivate ESM participants is to establish a “research alliance, in which the participants understand the importance of their contribution to the study” [36]. The recommendation can be applied in a company when talking to employees about ICC. Generally, insight from ESM can help design an ICC system, especially when it comes to user experience. The strong standing of ESM and its structural similarity to ICC hint at prospects for ICC adoption.

### 6.5. Persuasive Technologies

Persuasive technology is about interactive computing systems designed to change people’s attitudes and behaviors [37,38]. Consider ICC sensing requests such as “Have you taken a break from the sedentary position within the last hour?” or “How many months ago did you change the password to your work PC?”. These are used to collect useful occurrence data on health-related working habits and on following security procedures. But apart from being a means to collect data, such requests also have a persuasive component. Employees who receive them are implicitly reminded that it is desirable (or required) to take a break from the sedentary position or change one’s password. The reminding happens even if a request is disregarded or dismissed. Reminders are among the top motivational strategies applied in persuasive technology [38,39]. Apart from being an implicit reminder, a sensing request implements a self-report strategy [39], where a user provides feedback to the system. In summary, a subset of ICC consisting of *persuasive sensing requests* (like those above) may be treated as a workplace persuasive technology aiming to implement a company’s procedures and policies. Such requests may lead to self-incrimination, so one should prioritize making the responses anonymous.

## 7. ICC as Organizational Process

In this section and the next one, we look at ICC from the organizational point of view. Here we outline a possible company-wide process of implementing and running ICC. We assume that a platform with the functionality of the ICC sub-system (Figure 2) is available, probably as a third-party product. It could include the request manager, user manager, and databases. For simplicity, we consider personal delivery only.

**Figure 4:**
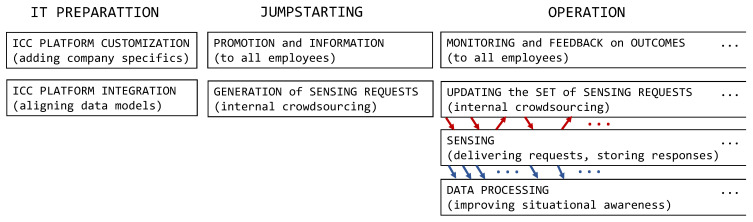
A possible ICC organizational process: three consecutive phases, each with concurrently running “threads.” The arrows represent adding or removing sensing requests and injecting responses to the context repository, respectively. Other inter-thread dependencies are not shown.

The process, shown in Figure 4, consists of consecutive phases, each with concurrently running, interdependent “threads.” The first phase, IT preparation, comprises customization and integration of the ICC platform. The customization starts with “telling” the platform about a company’s organization (divisions), the structure of its workforce (positions), and the structure of its space (assorted locations); it ends with populating the user database (Figure 2) with employees. The integration ensures interoperability with the company’s context repository or another data store. A data model translation plug-in to the ICC platform may be needed.

The second phase is jumpstarting. Here we have a promotion and information thread: advertising the new activity to the employees, providing a rationale, explaining how it works, describing incentives, presenting risks and possible downsides, and answering questions. The rationale may inform how data obtained with ICC will improve the company’s operation; this follows the recommendation to “identify and communicate the business value of data,” a step in creating a data-driven culture [40]. The downsides may include a negative impact on the employees’ privacy or an incriminatory flavor of some sensing requests. This, in turn, complies with the suggestion to “manage the ethical implications of data and analytics” [40]. Such a thread is an opportunity for the management to demonstrate its commitment to ICC—an element much needed in ICT technology adoption [41].

The other jumpstarting thread generates an initial set of sensing requests. This can be done by asking the employees themselves, via an internal crowdsourcing campaign [42] initiated with an open call for sensing request proposals. Thus, everybody, not just the management, can contribute to setting up ICC. Such involvement of the workforce may reveal things that the employees see as problematic or unknown. It may also contribute to ICC being accepted, once it is operational. The proposals undergo some curation before they are stored in the request database (Figure 2).

The third phase, operation, comprises four threads (Figure 4). The sensing thread, essential to ICC, consists in delivering sensing requests to the employees, collecting responses, and injecting them into the context repository. The sensing thread is realized by the ICC sub-system (Figure 2). The updating thread injects new sensing requests to the request database and removes old ones from there, on the fly. A sensing request may be removed when it is no longer needed or when it is not well received by the employees (as detected by the monitoring and feedback thread described below). A new sensing request may be proposed by any employee at any time; in fact, the internal crowdsourcing of sensing requests, started in the jumpstarting phase, may continue in the operation phase. Adding a new sensing request, and getting first responses to it, may happen very quickly. The data processing thread is where assorted applications (data analytics algorithms) derive higher-level insight from data in the context repository. Moreover, some raw data may be presented directly to responsible personnel. While not specific to ICC (data from different sources are processed together), the processing thread is where ICC adds value.

The monitoring and feedback thread serves two distinct (though related) functions. First, it assesses the level of employee cooperation with the ICC system (e.g., whether the employees respond, whether there are signs of fake responses, etc.). The thread regularly calculates indicators that capture the “health” of the ICC system in this respect. This should not be done for an individual employee but for the workforce at large. Regularly assessing and inspecting the indicators amounts to runtime monitoring of the user acceptance of ICC.

A simple acceptance indicator would be the overall response rate: the ratio of the number of all sensing request deliveries to the number of deliveries responded to. The response rate could also be maintained for each sensing request, to detect those requests that are frowned upon by employees. Another per-request indicator could measure inconsistencies among responses obtained when the oversampling factor is greater than one. A high level of inconsistency would suggest fake or incorrect responses.

Disturbing values of the acceptance indicators should not lead to any actions against employees. Overall indicators require a big picture outlook. A low overall acceptance may indicate that the promotion and information thread was less than perfect; it may also point to a broader problem rooted in a dysfunctional organizational culture. A disturbing value of a per-request indicator is easier to deal with; the sensing request in question could be modified or removed. Beyond that, the mere fact that a sensing request “failed” may lead to some insight.

The other function of the monitoring and feedback thread is to gauge benefits from ICC and inform both the management and employees on achieved positive outcomes. The regular feedback may include collected data, the company’s performance indicators, or actions taken. For example, one could highlight the amount of saved energy. Pinpointing the benefits may be difficult, but it should be done to keep employees motivated. The operation phase, and thus the entire ICC process, has no pre-determined end. It continues indefinitely, with the set of sensing requests updated on the fly to fit current needs.

Notably, in our ICC process, employees do not simply participate in the sensing thread by responding to sensing requests. They also contribute the requests via an on-going internal crowdsourcing campaign. Thus, there are two kinds of active participation in ICC, open to all employees: task execution and ”sharpening … automated routines in the organization, such as deciding on variables to be monitored” [1]. To ensure participation, employees receive comprehensive information about the process and its outcomes.

## 8. Further Organizational and Managerial Outlook

In this section, we consider a few selected perspectives. They include datafication, pulse surveys, customer feedback devices, internal crowdsourcing, organizational culture, and reasons to participate.

### 8.1. Datafication

Today, data is a part of a company’s capital [43]. This leads to a call to datafy a company’s activities, i.e., to capture data on them as they happen [43,44]. Datafication is usually achieved via “an application, device, or sensor” (e.g., an IoT device) embedded into an activity [43].

ICC can be used to datafy activities, places, or objects, wherever and whenever employees are present. This makes it a general-purpose datafication technique. ICC is unique in that it does not require applications, devices, or sensors embedded in a datafied target; the roles of these technical additions are played by humans, who act as multipurpose sensors. At the same time, ICC is susceptible to general criticism towards datafication, namely that it leads to surveillance and loss of privacy [44,45].

### 8.2. “Instant” Input Collecting

Here we consider company-run systems that prompt people (employees or not) to enter simple pieces of information. Such systems (a) work in the pull mode, i.e., take initiative, (b) prompt on a regular basis, and (c) ask for little time and attention (they are “instant”). ICC is meant to satisfy all these conditions. The following input-collection activities have become popular, which we treat as an indication that ICC itself is feasible.

Pulse surveys are minimalistic surveys administered to employees to track issues “in real time” [46,47,48]. Load balancing is applied when asking people to participate [48]. Pulse surveys focus on the employees’ sentiment, satisfaction, engagement, attitudes, motivation, or work experience, and thus belong mainly to the domain of human resources [49]. Unlike that, ICC is about plain facts. The difference in objectives leads to different time scales: months for pulse surveys vs. weeks, days, or even hours for ICC.

Customer feedback devices allow customers to declare a level of satisfaction with a service (see, e.g., [50]). These standalone units feature a minimalistic and intuitive user interface. We consider them working in the pull mode, as their mere presence and ease of use can be interpreted as a prompt. Makers envision them to be used, not only by “generic” customers, but also by patients, students, visitors, passengers, and even employees [50]. Our POI terminal is much like a customer feedback device, except they acquire data of different kinds. A device maker proposes asking employees about “happiness rating, wellbeing monitoring, engagement levels, change management, employee concerns, and culture alignment“ [50]. ICC collects plain facts, not opinions. On top of that, ICC includes personal delivery and role-targeted requests, both not possible with a customer feedback device.

### 8.3. Internal Crowdsourcing

Internal crowdsourcing [42,51] is, in simple terms, outsourcing to an organization’s own employees. It has been more formally defined as “an IT-enabled group activity based on an open call for participation in an enterprise” [42]. ICC does not fit the definition in that it is not driven by an open call, but by multiple, assorted sensing requests. Furthermore, the “crowd” in internal crowdsourcing is flat, while in ICC it is structured (which gives rise to role-targeted requests). Therefore, ICC is not a group activity; responding to a given sensing request may be limited to just one employee. ICC fits the definition in that it is IT-enabled and limited to the employees. To offer a more detailed comparison, we review the components of a conceptual framework for internal crowdsourcing proposed in [42]. The framework applies to ICC at the component level; differences are seen inside individual components.

The two components of the framework where the differences are most fundamental are: problems to be addressed and expected outcomes. For internal crowdsourcing, the problems are in the areas of collective intelligence, design, and decision making; the outcomes are, respectively, knowledge integration, innovation, and choices [42]. These challenges are typically of substantial intellectual weight and may include developing new product ideas or business strategies [51]. For ICC, the problems and outcomes are more down-to-earth: the problem is the lack of some data about an existing activity, place, or object, and the outcome is occurrence data about that entity. In addition, while an internal crowdsourcing open call usually targets a single challenge, ICC is multi-topic; sensing requests may belong to different areas (Table 1).

As to the governance component, the framework lists issues, such as corporate culture, incentives, quality assurance, and employee community management [42]. Both internal crowdsourcing and ICC are different from the usual hierarchy-based work, and they need changes in the management model. Thus, governance issues are relevant to ICC, and some governance solutions may work well for both techniques.

Another component of the conceptual framework covers roles that people play in internal crowdsourcing [42]. Requestors initiate and manage a campaign, while solvers contribute their knowledge and ideas. These roles have their counterparts in ICC, but the respective tasks are different. Internal crowdsourcing requires a solver to expend significant effort to come up with new ideas. According to [51], “given their other work responsibilities, many employees do not have time to participate in crowdsourcing activities”. In ICC, a “solver’s” involvement is limited to a short reaction to a sensing request, which, by definition, should be easy to respond to (although, without proper load balancing, interruptions may occur too often).

The next component is the use of IT [42]. Internal crowdsourcing needs a platform that facilitates collaborating and exchanging ideas, e.g., idea management software. Unlike that, ICC involves a high level of automation and integration with existing IT infrastructure. In ICC, possibly sophisticated algorithms manage requests and users. Integration with a pre-existing data model is needed to merge data from ICC with data from other sources. In terms of the layers identified in [1], a technology for internal crowdsourcing belongs to the group and community layer, while that for ICC to the intelligent augmentation layer. The ICC sub-system fits the description of intelligent augmentation in that it is “able to perform managerial roles” and “produce work activities for humans” [1].

The last component of the conceptual framework is the process. For internal crowdsourcing, it is a complete project cycle, with a beginning and an end, and with sequentially ordered phases [42]. Unlike this, our ICC process (Figure 4) has no specific time limit, and the “threads” run in parallel. Furthermore, in ICC, outcomes (occurrence data) are available from the very beginning of the operation phase.

### 8.4. Organizational Culture

Here we offer some suppositions on the interplay between ICC and organizational culture. We argue in support of our statements, but they need to be verified experimentally.

A pre-existing company culture may be conducive to ICC, or it may be an impediment to it (this has been observed of information systems in general [41]). A basic prerequisite for employee cooperation is a non-adversarial relationship between a company and its workforce. For example, a company that applies extensive and strict employee surveillance is likely to fail in adding ICC to its already intimidating data collection routines.

A culture of candor [52] seems to be a conducive one. It focuses on transparency, which is “the degree to which information flows freely within an organization, among managers and employees” [52]. The way to transparency is through “an organizational climate in which no one fears the consequences of speaking up” [52]. For some sensing requests, ICC relies on the lack of fear of “speaking up”; thus, a culture of candor should ease the acceptance of ICC.

Notably, there seems to be an impact in the opposite direction as well; ICC, if introduced successfully, may affect a company’s culture. It has been observed that information systems can positively contribute to culture [41]; new digital/human work configurations may lead to deep, “third-order effects” [1]. Here, we hypothesize four ways this can happen in the case of ICC.

As pointed out in [53], technology plays a significant role in the emergence of transparency in its many forms. ICC, yet another channel of information flow within a company, contributes to transparency by prompting small but concrete actions (i.e., responding to sensing requests). Practicing transparency within ICC may inspire people to expand it and thus get closer to a culture of candor.

ICC is non-hierarchy-based work, just like internal crowdsourcing [42]. The latter starts with an open call, directed to all, independently of their positions. And, ideally, the employees of a company contribute as equals. As such, internal crowdsourcing may trigger a shift towards a more democratic culture [41]. ICC is egalitarian as well. While with role-targeted sensing requests, different people are asked about different things, the key use case of handling a sensing request is the same for all. Therefore, one may argue by analogy, ICC should also lead to democratization. By participating without hierarchy-based differentiation, employees may increasingly see themselves as active agents in their company.

Responding to a sensing request makes an employee focus, even if very briefly, on the topic of the request. Occasionally having specific workplace activities, places, or objects at the center of attention, an employee may become inclined to see, so far overlooked, things without a prompt. In fact, ICC could be a tool to achieve just that: make employees more attentive to some “critical cues” and thus more aware of what is happening around them (e.g., noticing that lights are unnecessarily on). Company-wide perception, the first level of situational awareness [54], should then improve. For an employee, noticing things may trigger a sense of responsibility and engagement (e.g., turning a light off).

Finally, ICC may contribute to a data-driven culture by involving employees in data acquisition and demonstrating benefits obtained from the data, e.g., reducing energy consumption. Having continuous feedback on outcomes, employees learn that “their” data make a difference. Then, one can likely conclude that any data can be of value.

### 8.5. Motivation to Participate

We describe ICC as an extra tool, orthogonal and complementary to existing work done by employees. As such, participation in ICC is not a part of job descriptions. Responding to sensing requests does not lead to gains in one’s individual job performance; hence, performance expectancy [55] cannot be counted on as a factor leading to participation. As ICC is, by definition, voluntary, the same conclusion applies to social influence [55]. On the positive side, effort expectancy [55] should work for ICC, which is designed to require minimal effort. Overall, ensuring widespread employee participation appears non-trivial.

Incidentally, the same problem is researched for internal crowdsourcing, without clear conclusions [42]. As contributions in ICC are not nearly as prominent as those in internal crowdsourcing (e.g., new product ideas), company-wide “fame” is not a viable incentive in ICC. Further, recognizing employees active in ICC would likely put pressure on less active ones, which would violate the principle of voluntary participation.

Some people may be motivated by outcomes they value at the personal level. Energy savings enabled by ICC could motivate environmentally conscious people. Organizational citizenship behavior (OCB) may also play a role. According to the original definition, OCB refers to an employee’s behavior that “is discretionary, not directly or explicitly recognized by the formal reward system, and that in the aggregate promotes the effective functioning of the organization” [56]. Responding to an ICC sensing request satisfies these conditions perfectly. The extent, to which ICC may rely on organizational citizenship behavior certainly depends on how prevalent the latter is in the company (which leads us back to the issue of culture).

Incentivization has been researched in the context of mobile crowdsensing and the ESM method, among others. For example, adding personalized feedback and gamification to ESM is examined in [57] and [58], respectively, and in [59] a survey of incentives for mobile crowdsensing is presented. Some results from this research may apply to ICC. However, compared to “public” crowdsensing, ICC presents a more subtle challenge. In the public case, the relationship between a participant and the organization is based on recruitment for crowdsensing tasks alone [59]. The relationship is much weaker than in the case of ICC, where it is based on employment. Unlike in the public case, the pool of ICC participants, i.e., the workforce of a company, is not easy to change. Organizational culture, mutual dependence between an employee and their employer (where ICC plays a marginal role at best), and the possibility of having more privacy-sensitive sensing requests (than, e.g., a request to report traffic intensity in a city) all make a difference. The unique context of a company, with all its complexities, may call for a fresh look at incentive mechanisms.

## 9. ICC Evaluation Survey

In this section, we present feedback on ICC from people employed at different organizations. In the survey, we focused on acceptability, to determine if employees can coexist and cooperate with an ICC system. We also asked about valuable data that can be acquired, as well as possible problems or threats.

### 9.1. Online Questionnaire

The survey was conducted as a computer-assisted web interview (CAWI). We started with a short pilot phase and noticed that a number of pilot respondents had mistakenly understood that ICC covered not just plain facts, but also opinions or interpretations. To avoid the confusion, we explained the concept more clearly in the introduction to the questionnaire and added reminders to some of its questions. It took about fifteen minutes to complete the revised questionnaire. We used non-probabilistic, convenience sampling. An invitation to the survey was distributed via the newsletter of the Centre for Innovation and Technology Transfer Management at Warsaw University of Technology as well as via social media. We stressed that we were seeking respondents with work experience.

The final version of the questionnaire is shown in Table 2. In the introduction, we present an imaginary ICC application named INFOBot (without using the term “intra-company crowdsensing”), which acquires data that employees know but never report (Table 2, item 1). We list energy consumption in the office, security, and decision-making as areas, in which the data might be of value. Next, we provide four principles of interacting with INFOBot (Table 2, item 2) and ask a respondent to imagine being contacted by it (Table 2, item 3). We also ask the respondent to “put themselves in the shoes” of an employee who could receive sensing requests included in the questionnaire.

In the next section of the questionnaire (Table 2, items 4–8), the respondent is presented with five sensing requests, formatted as if they were INFOBot messages. To keep the wording simple, we refer to the requests as “questions.” For each request, the respondent picks a Likert scale level, indicating how likely they would “answer the question.” If the respondent’s choice indicates a problem, we ask for an explanation.

In the subsequent section (Table 2, items 9–13), we ask for a high-level evaluation of the INFOBot concept: how many sensing requests per time unit the respondent would be willing to receive, whether they would be open to having INFOBot in their company, and if INFOBot can have a negative impact on their comfort at work (and if so, in what way). Finally, we ask the respondent about useful data that INFOBot can acquire, and any potential INFOBot-related problems, limitations, or threats. In the last section of the questionnaire (Table 2, items 14–21), we ask for the respondent’s demographic data.

### 9.2. Respondents

A total of 122 respondents completed the questionnaire, approximately 50% of them female. The distribution of the respondents among the age groups was as follows: 23% in the range 18–25, 43% 26–35, 30% 36–45, 3% 46–55, 1% 56–65, and 0% in the range 66 or more; almost 70% of the respondents were not older than 35. Companies of different sizes, from microenterprises to big organizations, were represented relatively uniformly. The distribution of the respondents among companies with different numbers of employees was as follows: 19% in the range 1–9, 24% 10–49, 19% 50–249, and 35% in the range of 250 or more; some respondents did not know the size of their company. There was a wide variety in the functions of company divisions (with around 30 categories represented) and job titles (although most referred to knowledge work). Approximately 33% of the respondents managed people; for these, the average and maximum numbers of direct and indirect reports were 10 and 70, respectively. As to stationary vs. mobile work, the largest group were desk workers (71%), followed by those who do both (22%) and those who mostly move at work (7%). Apparently, a significant majority of our respondents do office work (as opposed to, e.g., construction or manufacturing). While we did not ask about nationality, we have every reason to believe that the respondents were Polish or worked in Poland.

### 9.3. Results

**Figure 5:**
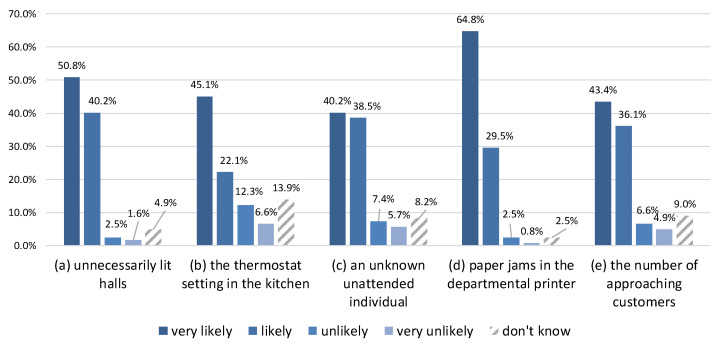
The willingness to answer the five sensing requests included in the questionnaire.

There were no statistically significant differences in terms of the influence of age or gender on the acceptance of ICC. Generally, the participants declared willingness to respond to the presented sensing requests. The percentages for the Likert scale are shown in Figure 5. As many as 91% of the respondents stated that they would very likely or likely answer the question about unnecessarily lit halls (Figure 5a). One explanation given for negative replies was information overload and the need to ignore insignificant stimuli from the environment.

For the thermostat setting in the kitchen room, approximately 67% of the respondents said they would be very likely or likely to answer (Figure 5b). This sensing request requires more involvement from an employee: going to the kitchen, locating the thermostat, checking it, and coming back. Most of those who reported they would be unlikely or very unlikely to respond to the request mentioned the extra burden. Some people said they never paid attention to such devices or did not know how to check the setting.

Regarding the sensing request about having seen an unknown, unattended individual on the company’s premises (implying a potential security threat), approximately 79% of the respondents declared they would be very likely or likely to answer the question (Figure 5c). Most of the others raised the problem of not knowing all the employees. One person mentioned a possible violation of the outsider’s privacy. Another was afraid of getting involved in a time-consuming investigation by building security. Some people did not like the idea that responding to a security-related sensing request was not mandatory.

The sensing request about paper jams in the departmental printer was accepted almost without reservations. As many as 94% of the respondents declared that they would be very likely or likely to answer the question (Figure 5d). However, one person mentioned information overload and the desire to ignore everything inessential. Another person said that the issue was just too trivial to remember.

The last sensing request had to do with the number of customers who approached an employee (presumably a salesperson in a store) asking for help. Approximately 79% of the respondents said that they would be very likely or likely to answer the question (Figure 5e). The skeptics were mainly afraid that such requests would serve to evaluate an employee. Some respondents stressed the need for anonymity.

**Figure 6:**
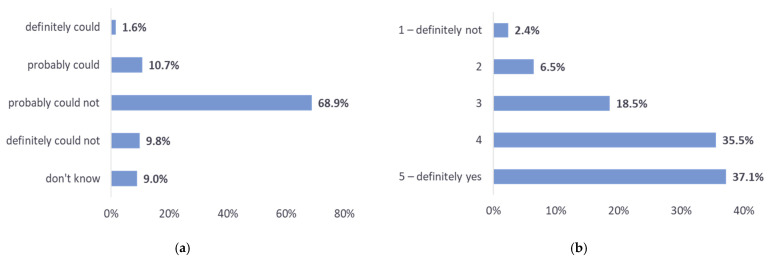
Opinions on INFOBot. (**a**) Could it have any negative impact on one’s comfort at work? (**b**) Would one be open to having it in one’s company?

Having seen our introduction to INFOBot and sample sensing requests, the respondents gained some understanding of the concept of ICC. Then they were asked to provide an overall evaluation. Two of the evaluation questions were focused on ICC being accepted by employees. We asked if INFOBot could negatively impact one’s comfort at work (Figure 6a). Almost 80% of the respondents replied, “probably could not” or “definitely could not”. Those who were afraid of reduced comfort (13%) mentioned the following reasons: being interrupted, having to report on the quality of one’s work (or that of one’s colleagues), and having an additional task to perform. We also asked if a respondent would be open to having INFOBot in their company. As many as 72% of the respondents replied “yes” or “definitely yes”, while only 9% percent replied “not” or “definitely not” (Figure 6b).

**Figure 7:**
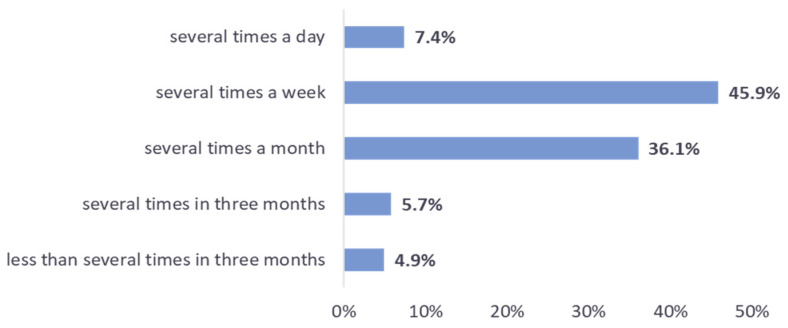
The willingness to receive several INFOBot questions in a given amount of time.

While the respondents were generally open to having INFOBot at work, they would not like to deal with too many sensing requests in a short time. Only 7% of them replied that they would be willing to receive several INFOBot questions a day. The number of those willing to receive several questions per week, month, and three months, were 46%, 36%, and 6%, respectively. About 5% preferred to receive less than several questions per three months (Figure 7).

Regarding valuable data to be acquired with ICC, the respondents mentioned equipment faults, energy usage (e.g., devices unnecessarily turned on), availability of supplies (e.g., paper, coffee, detergents), tidiness at the workplace, work safety, good work habits, security, and cybersecurity. We transformed some of those proposals into several sensing requests included in Table 1. Notably, even though we explicitly reminded the respondents that ICC is about plain facts, a number of them replied that INFOBot could collect employees’ opinions on assorted topics.

Finally, we asked about problems, limitations, or threats related to INFOBot. We collected replies to that question, as well as remarks dispersed elsewhere in the completed questionnaires. Then we compiled a list of issues with ICC, as perceived by the respondents:

1. Disruption. One more source of interruptions, while being overloaded and targeted by too many existing sources of interruptions. The need to pay attention to one’s environment under information overload. Having an extra task.
2. Self-incrimination, acting against one’s interests. A “big brother” technology to control employees. The need to respond with care to avoid self-incrimination. Not trusting the system (e.g., about effective anonymization of responses). “Exaggerated” savings resulting from insight acquired with ICC.
3. Lacking a rationale to contribute, if there is no concrete information on the goals and effects of ICC or no follow-up actions.
4. Demotivating routine. Getting bored with responding to the same sensing request(s) repeatedly, especially if the problems addressed by those requests do not exist. Responding mindlessly to get rid of a sensing request. Ignoring the system.
5. Not knowing how to answer. Unrealistic expectations as to what people should know. Reluctance to inspect unfamiliar technical devices. Being asked about something one considers unimportant or too trivial to remember.
6. Gaming the system (providing false responses to manipulate). Conspiring to avoid a change or to get desired outcomes.
7. Getting in legal or procedural trouble (e.g., getting involved in an inquiry). Violating somebody else’s privacy.

Our respondents also contributed constructive proposals as to how ICC should work. Some of them fully comply with our concept of ICC. These include (a) the need to clearly communicate the goals and results of ICC, including tangible outcomes (e.g., actions taken), (b) context-aware delivery configurable by an employee (e.g., setting the time to allow focus hours), (c) delivering to the right people via role-targeted requests, to avoid bothering people who do not know how to answer, (d) allowing employees to contribute sensing requests, and (e) preserving anonymity, if possible.

Other constructive proposals go beyond our concept of ICC.

1. Allow opinions, not only facts. The opinions may be about the ergonomics of a chair, work overload, satisfaction, mobbing, or evaluation of new initiatives. Allowing opinions would lead to a hybrid solution, with a flavor of experience sampling or even pulse surveys. As people seem eager to contribute opinions, making the system hybrid may ease its adoption.
2. Add a practical hint to a sensing request. For example, a sensing request about paper jams might include contact information for a technician. A sensing request delivered to a new employee might be augmented with a tip to ease the onboarding process. The hint could be retrieved with a “get hint” button added to the request interface.
3. Use ICC-triggered interaction opportunities to obtain unsolicited data. When an employee responds to a sensing request, they could contribute a piece of data or idea not necessarily related to the topic of the request. An “add comment” button could serve the purpose.
4. Occasionally offer a reward for participation.

### 9.4. Conclusions

We consider the results shown in Figure 6 and Figure 7 to be quite encouraging. If we define a positive evaluation of ICC as one in which a respondent replies that (a) INFOBot probably or definitely could not negatively affect their comfort at work, *and* (b) they would be (or definitely would be) open to having INFOBot at work, *and* (c) they would be willing to receive *at least* several sensing requests a month (i.e., several requests per day, week, or month), then the fraction of positive evaluations was approximately 63% of the total. The positive evaluations outnumbered others by a wide margin.

At the same time, both those who gave the positive evaluations and those unconvinced made insightful comments with reservations and proposals; the major ones are listed above. Some recurring themes from the respondents coincide with our own concerns; apparently, successful implementation of ICC requires careful thought. Here we provide tentative recommendations derived from the survey.

1. Apply conservative sensing request rate control and load balancing policies. Do not overload. Perhaps a user should be able to indicate that the current load is too high (e.g., with a “too many” button added to the interface of a sensing request). Such feedback would amount to personalized rate control following individual tolerance levels. Consider delivering sensing requests at “low intensity” times at work, e.g., at lunchtime.
2. In the load balancing algorithm, consider both the number of sensing requests delivered to one employee and their diversity. If possible, vary the requests to fight routine and boredom.
3. Be careful with sensing requests that (a) require a recipient to move to a location and back (instead, consider a POI terminal), (b) require recalling something observed earlier, or (c) call for interaction with technical infrastructure (people may not be comfortable interacting with devices, no matter how simple they appear to be).
4. Make it clear that ICC, being voluntary, is not meant to reliably detect *every* undesirable occurrence, no matter how dangerous (e.g., security-related). Instead, ICC should reveal that undesirable things do happen, and corrective action is needed to prevent them from happening in the future.
5. Consider adopting a “generalization policy” of handling data from those sensing requests that may lead to (self-) incrimination or loss of privacy (Section 4). The generalization policy would be an explicit, company-wide commitment to treat such sensitive data as symptoms of generic (systemic) problems and not as evidence in individual cases. (In [60], P. Drucker points out that an individual occurrence is most often just a symptom and should be treated by seeking a generic solution). In addition, the policy ensures that responding to a sensing request never leads to a case-specific inquiry or procedure in which a responding employee needs to participate.
6. Consider adding a rationale to a sensing request. The rationale should explain why the request is needed or what benefits result from responding to it. The rationale could be retrieved with a “why ask” button.

A commonly recurring theme, which we both predicted and confirmed via the survey, is the concern that ICC may become a tool to monitor the workforce, used, in the end, against responding employees or their colleagues. We have commented on the problem in several places, pointing to “neutral” sensing requests, anonymity, raising the level of abstraction of acquired data, understanding the benefits of internal whistleblowing when it comes to grave issues, a culture of candor, and a generalization policy.

**Figure 8:**
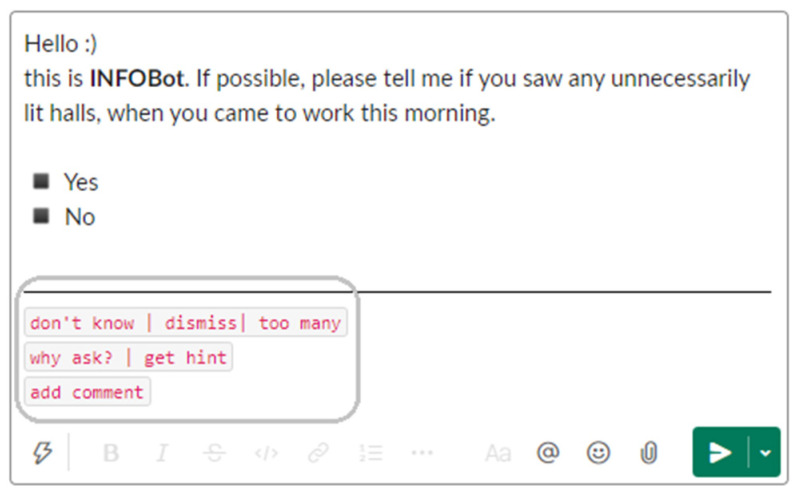
Possible rendering of a sensing request, with the full selection of generic buttons (enclosed); based on Slack [10].

We conclude this section by presenting a sensing request with all the previously mentioned additional buttons (Figure 8). The buttons below the horizontal line are generic, i.e., present in every request. The three buttons in the first row allow a user to quickly offer some “housekeeping” feedback without responding to the request. The two buttons in the second row are a way to help an employee stay motivated and informed when it comes to ICC. Viewed differently, the “why ask” and “get hint” buttons are a means to disseminate information about a company’s objectives, policies, regulations, or operating procedures, piece by piece and in the context of related sensing requests. Finally, the “add comment” button adds a feature of opportunistic interaction: whenever a sensing request is delivered, an employee has a chance to write anything on whatever topic, whether related to the request or not. With these buttons, an ICC system supports both intra-company crowdsensing and wider intra-company communication.

## 10. Summary and Future Work

This paper defines occurrence data as company-relevant plain facts an employee learns simply by doing their job. We stressed that, until now, a lot of occurrence data, while potentially valuable, have been overlooked. We proposed and elaborated the technique of intra-company crowdsensing (ICC), a way to extract occurrence data from employees. In doing so, we approached ICC from different points of view: user-centered (the interaction model), technical (the system architecture), and organizational (the ICC process). We positioned ICC against the background of information and communication technologies (ICT) as well as selected managerial issues. Finally, we conducted a survey to learn how employees react to the concept of ICC. The results indicate that ICC is worth pursuing, but one should design an ICC system with care for it to be accepted.

This paper provides groundwork and calls for further research. One key research challenge is to explore the nature, scope, and value of occurrence data. A technique to extract occurrence data is justified only if the data itself is valuable. Attention should be paid to non-office work environments, as the present paper tends to focus on the office domain. Another key challenge has to do with ICC being accepted by the workforce. The conditions of benevolent employee participation, when an employee responds and provides correct data, seem more subtle than in the case of “public” crowdsensing. There are implementation challenges related to modeling an organization, developing algorithms to handle sensing requests and manage users, as well as integrating ICC with pre-existing IT infrastructure. Our next step is to implement an ICC system, deploy it in a company, and validate ICC via an experiment in-the-wild. We also hope that this paper will trigger interest in ICC in the research community.

ICC is not a datafication panacea. However, we believe that, in *every* organization, at least *some* highly useful sensing requests, acceptable to its employees, can be found.

## Figures and Tables

**Table 1:** Examples of sensing requests in different “application areas”.

**Energy and Water Conservation**
Did you see any unnecessarily lighted halls when you arrived at work this morning?
Are there any open windows in the hall next to your room?
What is the thermostat setting in the kitchen room?
Are the blinds in your room open or closed?
Have you seen any water leaks in public toilets today?
**Customer Intelligence**
Approximately how many times has a customer approached you within the last two hours? (*A supermarket salesperson*).
At which department, shoes or accessories, are there currently more customers? (*A salesperson at a department store*).
Approximately how many customers asked about “The Books of Jacob” today? *(A bookstore*).
Is your bus crowded now? (*A public transport bus driver*).
Which sculpture attracted most attention from visitors today? (*A gallery attendant*).
Did the passengers on your last flight to Budapest have a hard time finding space for their cabin luggage in the overhead compartments? (*A flight attendant*).
Was your last customer a single driver or were there more people in the car? (*A drive-through restaurant*).
Approximately how many students attended your COMPUTING 101 lecture earlier today? (*A university instructor*).
Approximately how many patients arrived improperly prepared for the spirometry examination today? (*A spirometry nurse*).
**Security and Cybersecurity**
Have you seen any unattended individual, unknown to you, on the premises today?
How many months ago did you change the password to your work PC?
Have you seen any unattended computer with a user logged on today?
Have you seen any laptop with the camera not covered up today?
**Occupational Safety, Accident Prevention, and Work Habits**
Can you see any dangerous tools left unattended? (*A factory*).
Have you drunk one liter of water today? (*A factory, near hot machinery*).
Have you seen a wet floor today? (*A factory*).
Is there an injury warning sign next to your machine? (*A factory*).
Can you smell a chemical now?
Did you see anybody not wearing a helmet at the construction site yesterday?
Have you seen any loose object, which could fall from the scaffolding? (*A construction site*).
Have you taken a break from the sedentary position within the last hour? (*Office work*).
**Work Organization and Sociometry**
How many participants were there at your last meeting?
How long was the last meeting you participated in?
Did your last meeting end with action items?
Approximately how much time did you spend handling e-mail yesterday?
Have you talked to anybody from a different department within the last two weeks? (*Sociometry*).
How many assignments due the next day have you received this month?
**Access to Facilities, Infrastructure, and Supplies**
Approximately how many times have you experienced paper jams in the departmental printer within the last thirty days?
Are all lights in your area operational?
Have you had to wait for a parking space within the last seven days?
Have you experienced a lack of any office supplies this month?

**Table 2:** The ICC evaluation questionnaire.

**Introduction**	
1.	Rationale: employees know potentially useful facts that are overlooked …
2.	How INFOBot works: (a) It asks about plain facts or data; you don’t have to express opinions or make interpretations. (b) It asks about things you know simply because you do your work at the company. (c) Responding to INFOBot’s question should not take you more time than answering a typical SMS. (d) You can ignore INFOBOT’s question without any consequences.
3.	Imagine a typical day at work …
**Examples of Sensing Requests**	
Below (items 4–8), the responses marked with (i), (ii), etc., belong to the sensing requests themselves. To keep the wording simple, we refer to the sensing requests as “questions”. For each sensing request, a respondent is asked “How likely would you answer the question (or a similar one)? (a) very likely, (b) likely, (c) unlikely, (d) very unlikely, (e) don’t know.” If the respondent chooses “unlikely” or “very unlikely”, we ask for an explanation.	
4.	*Hello, this is INFOBot. If possible, please tell me if you saw any unnecessarily lit halls when you arrived at work this morning. (i) yes, (ii) no, (iii) I don’t remember.*
5.	*Hello, this is INFOBot. If possible, please tell me the current thermostat setting in the kitchen room.* *(i) 0, (ii) 1, (iii) 2, (iv) 3, (v) 4, (vi) 5.*
6.	*Hello, this is INFOBot. If possible, please tell me if you’ve seen any unattended individual, unknown to you, on the premises today. (i) yes, (ii) no, (iii) I don’t remember.*
7.	*Hello, this is INFOBot. If possible, please tell me approximately how many times you have experienced paper jams in the departmental printer within the last thirty days.* *(i) 0, (ii) 1–5, (iii) more than five times.*
8.	*Hello, this is INFOBot. If possible, please tell me, approximately, how many times a customer has approached you within the last two hours*.
**Concept Evaluation**	
9.	How often would you be willing to receive INFOBot questions, like those above? (a) several times a day, (b) several times a week, (c) several times a month, (d) several times in three months, (e) less than several times in three months.
10.	Would you be open to having INFOBot in your company? 1, 2, 3, 4, 5 (1—definitely not, 5—definitely yes).
11.	Could INFOBot have any negative impact on your comfort at work? Pick one: (a) definitely could, (b) probably could, (c) probably could not, (d) definitely could not, (e) don’t know.
11a.	If your answer is (a) or (b), please explain why.
12.	What useful information could INFOBot acquire from employees?
13.	Can you see any problems, limitations, or threats related to INFOBot? If so, please share your thoughts with us.
**Tell Us About Yourself**	
14.	What kind of work you do? Pick one: (a) my work is mostly stationary (at a desk), (b) my work is mostly mobile (I move a lot), (c) I do both kinds of work approximately equally.
15.	What is your position (job title) at your company?
16.	Do you manage people? (a) yes, (b) no.
17.	If you do manage people, approximately how many reports (both direct and indirect) do you have in total?
18.	How many people work at your company? (a) 1–9, (b) 10–49, (c) 50–249, (d) more than 249, (e) don’t know.
19.	Name the primary function of the company division (not the entire company) that you work at.
20.	Your gender: (a) woman, (b) man, (c) other, (d) I refuse to answer.
21.	Your age: (a) 18–25, (b) 26–35, (c) 36–45, (d) 46–55, (e) 56–65, (f) above 65.

## Data Availability

The data presented in this study are openly available in FigShare at https://doi.org/10.6084/m9.figshare.18978428.
